# Endocannabinoids and Human Sperm Cells

**DOI:** 10.3390/ph3103200

**Published:** 2010-10-12

**Authors:** Annarina Ambrosini, Rosamaria Fiorini, Giovanna Zolese

**Affiliations:** Department of Biochemistry, Biology and Genetics, Marche Polytechnic University, 60131 Ancona, Italy; E-Mail: a.ambrosini@univpm.it (A.A.); g.zolese@univpm.it (G.Z.)

**Keywords:** endocannabinoids, human sperm cells, idiopathic infertility, oleylethanolamide, palmitoylethanolamide

## Abstract

*N*-acylethanolamides (NAEs) are naturally occurring signaling lipids consisting of amides and esters of long-chain polyunsaturated fatty acids. Usually they are present in a very small amounts in many mammalian tissues and cells, including human reproductive tracts and fluids. Recently, the presence of *N*-arachidonoylethanolamide (anandamide, AEA), the most characterised member of endocannabinoids, and its congeners palmitoylethanolamide (PEA) and oleylethanolamide (OEA) in seminal plasma, oviductal fluid, and follicular fluids was demonstrated. AEA has been shown to bind not only type-1 (CB1) and type-2 (CB2) cannabinoid receptors, but also type-1 vanilloid receptor (TRPV1), while PEA and OEA are inactive with respect to classical cannabinoid CB1 and CB2 but activate TRPV1 or peroxisome proliferator activate receptors (PPARs). This review concerns the most recent experimental data on PEA and OEA, endocannabinoid-like molecules which appear to exert their action exclusively on sperm cells with altered features, such as membrane characteristics and kinematic parameters. Their beneficial effects on these cells could suggest a possible pharmacological use of PEA and OEA on patients affected by some forms of idiopathic infertility.

## 1. Introduction

Cannabinoids are a large number of different active compounds found in the marijuana plant (*Cannabis sativa*). It is well known that the primary psychoactive molecule in marijuana, Δ^9^-tetrahydrocannabinol (THC), has marked adverse effects for reproductive events in marijuana users (for a review see [1]). A family of unsaturated fatty acid derivatives, biologically synthesised by many tissues, has been termed “endocannabinoids” (EC), because they exert their effects acting as endogenous ligands for cannabinoid receptors. In the peripheral and neural tissues, they have been shown to modulate as paracrine or autocrine mediators, protein and nuclear factors involved in many physiological functions such as cell proliferation, differentiation and apoptosis [2]. *N*-arachidonoylethanolamide (anandamide, AEA) and 2-arachidonoylglycerol (2-AG) are the best characterized prototype members of two families of endocannabinoids, the fatty acid amides (NAEs) and the monoacylglycerols, respectively [3]. AEA belongs to the group of *N*-acylethanolamides (NAEs), which are naturally occurring hydrophobic molecules usually present in a very small amount in plants, invertebrate and in mammalian tissues [4,5,6]. Chemically NAEs can be considered as derivatives of a long acyl chain linked to the amine group of 2-ethanolamine by an amide bond. The content of long-chain NAEs and their precursor, *N*-acylphosphatidylethanolamine (NAPE), has been shown to increase dramatically in a variety of organisms when subjected to stress [7,8]. Recently, the presence of anandamide and its congeners palmitoylethanolamide (PEA), and oleylethanolamide (OEA) was shown in seminal plasma, oviductal fluid, and follicular fluids [9]. Furthermore, AEA was isolated in human spermatoza [10]. PEA and OEA are cannabinoid-receptor inactive molecules [11,12], with respect to the classical cannabinoid receptors CB1 and CB2 [13], which are G-protein coupled. 

PEA and OEA present some endocannabinoid-like effects, likely by the so-called “entourage effect” [14]. The term “entourage effect” was used for the first time to describe the ability of some 2-AG congeners to strength the actions of 2-AG at CB receptor [15]. However, it is also used to indicate the effects of PEA and OEA to potentiate the AEA actions at TRPV1 receptors [16]. This “entourage effect” can be performed by different mechanisms, such as the modulation of AEA target receptor [17], or the inhibition of endocannabinoids catabolism, thus increasing their levels to potentiate their biological actions [16,18]. 

Furthermore, increasing evidence indicates that OEA and PEA can themseles activate other receptors that are potential targets of endocannabinoids, such as TRPV1 (Transient Receptor Potential Vanilloid type (1) [14,19] or PPARs (Peroxisome Proliferator-Activated Receptors) [14].

## 2. Endocannabinoid System

This consists of endocannabinoids, enzymes involved in their synthesis and degradation, a putative membrane transport system and receptors through which they elicit their physiological functions [18]. Endocannabinoids are synthetised on demand from membrane precursors and are not stored [18].

NAEs are produced by enzymatic degradation of NAPEs. The major pathway is catalysed by a specific membrane-associated phospholipase D (NAPE-PLD) enzyme [20] which is regulated by a signalling mechanism [21]. *N*-Arachidonylethanolamide (AEA), *N*-palmitoylethanolamide (PEA) and *N*-oleoylethanolamide (OEA) are enzymatically released together from membrane phospholipid precursors when cells are stimulated by depolarizing agents, neurotransmitters and hormones [6,21,22,23]. NAEs are lipophylic molecules with a very low solubility in water [24], which are likely bound in biological fluids to albumin [24,25]. Moreover, recent data could suggest an interaction also with lipoproteins [26].

2-AG is synthetised by two enzymes: a specific phospholipase C (PLC), which hydrolyses inositol phospholipids generating diacylglycerol (DAG), and a *sn*-1-DAG lipase, which converts DAG to2-AG [3]. Degradation of NAEs requires their transport within the cell, but the transport mechanism is still debated. However, the presence of an endocannabinoid membrane transporter (EMT), was suggested for the uptake of extracellular AEA [10].

Once inside the cells, NAEs are quickly metabolized by a fatty acid amide hydrolase (FAAH), that breaks the amide bond and releases free fatty acid and ethanolamine [27]. FAAH is an integral membrane protein (located in endoplasmic reticulum) whose active site was supposed to be accessed by NAEs via the bilayer [28]. However a recent study identified a system of carrier proteins that transport AEA from the plasma membrane to FAAH in the rough endoplasmic reticulum [29]. Although PEA is hydrolysed by FAAH, it is preferentially demolished by the cysteine amidase *N*-acylethanolamine-hydrolyzing acid amidase (NAAA) [30,31] which is located in lysosomes [32]. 2-AG is degraded to arachidonic acid and glycerol by a specific monoacylglycerol lipase in the cytosol [33]. 

A recent study [10] demonstrated that endogenous AEA is present in human spermatozoa, together with the active AEA-synthase NAPE-PLD, the AEA-hydrolase FAAH and a purported carrier EMT. The possibility that OEA and PEA are co-released together with AEA by spermatozoa has been not yet examined. 

Cannabinoid receptors belong to the superfamily of G-protein-coupled receptors, producing an inhibition of adenylate cyclase activity and inhibition of calcium channel activation by depolarization [34]. Two main subtypes have been cloned and characterized. Cannabinoid receptor 1 (CB1) was originally cloned from rat and human brain [35,36]. Quite recent papers demonstrated that it is widely distributed in neural and nonneural cells in reproductive and other peripheral organs [9,37,38]. Functional CB1 receptors are expressed in male and feminine human reproductive tract [35,39,40,41,42,43,44,45,46], included human sperm [37,47].

Cannabinoid receptor 2 (known as CB2) was originally cloned from human promyelocytic leukemia HL 60 cells [48] and has important roles in modulating immune responses [22]. Functional CB2 receptors are expressed in some male and feminine tissues of human reproductive tract [39,41,43,49], but a functional CB2 receptor was not identified in human sperm [10], although it was isolated in porcine sperm [47]. It was demonstrated that CB receptors signalling, by differential activation of G-protein subtypes regulating multiple signal transduction pathways, can modulate capacitation and fertilizing potential of human and boar sperm *in vitro* [9,37,47,50]. AEA has also been shown to bind to the type-1 vanilloid receptor (TRPV1) [51] which is also expressed in human sperm, where it controls sperm/oocyte fusion [10]. 

More recently two G protein-coupled receptors (GPR) have been shown as novel cannabinoid receptors: GPR119 and GPR55. The former is activated by OEA and is strongly implicated in the regulation of energy balance and body weight, while the latter is activated by multiple different cannabinoid ligands, included PEA [13], and it is associated with pain signalling in wild-type animals [52]. The possibility that mammalian sperm also express these receptors remains to be investigated.

The localization of ECS components in human sperm was the post-acrosomal region for TRPV1, and the post-acrosomal region and the midpiece for NAPE-PLD, CB1 and FAAH [10].

ECS is involved in fertility not only in humans and mammalians but also in non mammalian vertebrates and invertebrates, being the system highly conserved from evolutionary view point [53]. Schuel *et al.* [54] were the first to show in the sea urchin *Strongylocentrotus purpuratus* that cannabinoids directly affect the process of fertilization by reducing the fertilizing capacity of sperm.

## 3. Endocannabinoids and Sperm Cells

A number of reviews on endocannabinoids and their effect on human reproduction have been published recently [3,9,18,50,55,56]. These reviews were focused on anandamide and another important endocannabinoid, 2-arachidonoylglycerol (2-AG). These endocannabinoids can perform multiple roles in reproductive tract fluids by modulating sperm motility, capacitation and acrosome reaction, regulating reproductive tract function, protecting against infection, and maintaining sperm viability [9,37,47]. Also recent studies have been focused on these molecules; e.g., Sun *et al.* [57] evaluated *in vivo* effects of sustained high AEA levels on sperm function. FAAH^−/−^ mice with high AEA levels were used as a model system to mimic conditions of long-term exposure to marijuana. Results with FAAH^−/−^ sperm have shown a compromised fertilizing capacity both *in vivo* and *in vitro*, and are clinically relevant because long term *in vivo* exposure to marijuana is implicated in reduced male infertility. Furthermore, Grimaldi *et al.* [58] have shown that 2-AG plays a pivotal role in the meiotic process during spermatogenesis by activating CB2 receptors, and, in mouse epidymis Cobellis *et al.* [59] have found a 2-AG gradient which regulates the activity of CB1 present on the sperm cell membrane, affecting spermatozoa motility. 

However, only few studies have been made on the human sperm effects of the congeners of anandamide, with endocannabinoid-like effects, that have been identified in the biological fluids of human reproductive tract, PEA and OEA [9].

This review will examine the present knowledge about the effects of these NAEs in human sperm cells. Ejaculated sperm of mammalians and humans, when bathed in male secretions included seminal plasma, are initially unable to fertilize oocyte [9,50,55,60]. Sperm cells can acquire fertilizing potential both *in vivo*, in the female genital tract or *in vitro*, following exposure to proper culture media [60,61]. The first step of fertilization process is called capacitation, that is the fundamental pre-requisite for ability of sperm to undergo acrosome reaction which enables it to bind to the egg’s zona pellucida and to fertilize it. One important aspect of the sperm capacitation is the remodelling of the glycocalyx on the plasma membrane which explains the dynamic alterations in lateral topology of the transmembrane proteins [62]. Capacitated sperm cells are characterised by hyperactivated motility (HA) necessary for fertilization. The molecular mechanism of capacitation and acrosome reaction, remain elusive [63]. It was suggested the occurrence of multiple signalling pathways that regulate sperm capacitation [64]. It is known that preparatory modifications, associated with both *in vivo* and *in vitro* capacitation, include multiple molecular changes in plasma membrane proteins/glycoproteins and sterols (mainly cholesterol) that modifies plasmalemma of spermatozoa [63]. Cholesterol efflux modifies plasma membrane physicochemical features such as membrane permeability and fluidity allowing the influx of ions, such as Ca^2+^ and HCO_3_^−^, starting a cascade of signalling events [63] including activation of adenylyl cyclase activity , production of cAMP, stimulation of PKA and likely other kinases, protein tyrosine phosphorylation [63]. A lower cholesterol-phospholipids ratio has been correlated with a faster capacitation time [65] in human sperm cells. 

Schuel *et al.* [50] showed that 0.25 nM R(+)-methanandamide (a stable analogue of AEA) stimulates sperm hyperactivated motility, that is inhibited by an higher concentration (2.5 nM) of the same compound. These biphasic responses are a general feature of cannabinoids and other G-protein coupled receptors, suggesting presuntive modulatory roles for AEA during sperm transport, capacitation and fertilization *in vivo*. 

Gervasi *et al.* [66] have demonstrated that bull sperm and bovine oviductal epithelial cells express CB1, CB2 and FAAH and that physiological concentration of AEA modulates sperm-oviduct interaction. By *in vitro* experiments with AEA, R(+)-methanandamide and CB1 antagonists, they speculate that the decrease in sperm binding to oviductal epithelium caused by AEA *in vivo* is mediated by CB1 receptors.

An interesting paper by Schuel *et al.* [9] reports that AEA, but also PEA and OEA, are significantly decreased in follicular fluid, with respect to seminal plasma and oviductal fluid, indicating that their concentrations are decreased when the sperm approaches oocyte to be fertilised. It was speculated that, the presence of AEA and the activation of endocannabinoid system in spermatozoa could be a mechanism to prevent sperm capacitation before the interaction with oocyte [56]. Our studies, focused on the effects of endocannabinoid-like PEA and OEA demonstrated that both molecules affect sperm parameters and as a consequence capacitation process [67,68,69]. 

Previous studies of men affected by idiopathic infertility demonstrated that spermatozoa from oligozoospermic men and some normozoospermic men (as defined by World Health Organization criteria) are characterized by plasma membrane alterations (decreased membrane polarity or increased membrane phospholipids packing) [70], which are likely related to altered *in vitro*-induced capacitation [68]. It was showed that idiopathic infertility is characterised by spermatozoa plasma membrane alterations [69 and ref. cited therein], that can be easily evaluated by the fluorescent features of the membrane probe Laurdan, that was suggested as simple tool for infertility assessment [70]. Data obtained by sperm samples from oligozoospermic patients and some normozoospermic men (threshold value of 20 × 10^6^ cells per milliliter for diagnosis of oligospermia) were shown in this paper. Spermatozoa from patients with< 32 × 10^6^ cells per milliliter were characterised by a markedly decreased membrane polarity (as measured by Laurdan fluorescence using the parameter defined as exGP^340^), with respect to spermatozoa from men with sperm cells> 32 × 10^6^ cells per milliliter [70]. Ambrosini *et al.* [68] demonstrated that cells with high exGP^340^ (Laurdan fluorescence) present physiologically decreased rate in the time pattern of plasma membrane capacitation processes, which could be related to their infertility. 

It was shown that 5 μM PEA affects the time course of *in vitro*-capacitation, increasing the rate of this process, in sperm cells from infertile men with high exGP^340^ [68]. This result brought up the authors to suggest the possible pharmacological use of this molecule in this pathology [68].

Also 2.5 nM PEA [67,68,69] significantly affect the capacitation process, as demonstrated by the PEA- and OEA-induced increase of some motility parameters and HA, that are involved in that complex mechanism. This effect was evident only in sperm cells characterised by low values of the kinematic parameters, such as VSL (straight-line velocity), VCL (curvilinear velocity), ALH (lateral head displacement) and LIN (linearity), whose modification is an important index for the evaluation of sperm capacitation [67]. When the capacitation process was induced in this group of spermatozoa, kinematic parameters are only slightly increased and no significant HA was measured [67]. In these groups of sperm cells, the capacitation process significantly increases kinematic parameters and HA percentage only when it is performed in the presence of 2.5 nM PEA [67]. PEA and OEA have no effect on sperm cells that are characterised by larger kinematic parameters, and by significant HA percentage in capacitating conditions. Although the mechanism involved in PEA and OEA action on these altered sperm cells remains to be elucidated, data obtained [67] demonstrated a PEA-induced increase of extracellular Ca^2+^-influx and could suggest a modulation of Ca^2+^-channels by this endocannabinoid-like molecule. 

In fact, an increase of free intracellular Ca^2+^ alters flagellar beat patterns characteristic of HA [71]. Moreover, the involvement of plasma membrane Ca^2+^-channels is thought to play a role in initiating and mantaining hyperactivation [72]. It was demonstrated that [60,73] Ca^2+^ and HCO_3_^−^ are involved in the regulation of SACY, an atypical adenylyl cyclase, that increases cAMP intracellular levels and activate PKA, that modulates tyrosyne phosphorilation involved in capacitation.

It is interesting to underline that *in vitro* incubation of normal human spermatozoa, in capacitating conditions, with 2.5 nM AM-356, an analogue of anandamide, inhibits HA [50]. It was suggested that this effect could be linked to a regulation of Ca^2+^-channels [50]. However, no modifications of intracellular Ca^2+^ concentrations were induced by AEA in human sperm during the sperm acrosomal reaction induced by the calcium ionophore ionomycin [37], ruling out any interfering effect of this endocannabinoid on Ca^2+^-signaling. 

PEA and OEA are not thought to be ligands of the cannabinoid receptor CB1, which is expressed in spermatozoa [50]. Although the action on TRPV1 and the “entourage effect” cannot be excluded, their chemical features suggest that their action can be performed, at least partially, through physical interactions with the lipid part of the cellular membrane. This possibility is suggested by previous papers that investigated the interaction of several NAEs, differing for acyl chain length and unsaturation, with dipalmitoylphosphatidylcholine (DPPC) multilamellar liposomes, by using fluorescence spectroscopy [74,75]. NAEs modified DPPC main transition temperature (T_m_) in different ways, depending on the characteristic of their acyl chains, suggesting that acyl chains length and unsaturation may play important roles in the NAE interaction with membranes. Zolese *et al.* [75] studied the effect of saturated and monounsaturated NAEs on DPPC large unilamellar vesicles and on porcine pancreas phospholipase A_2_ activity, which is dependent on lipid bilayer features. The data showed that the acyl chain length and the presence or the absence of a single double bond are crucial for the enzymatic activity modulation by NAEs, in particular PEA was shown to increase PLA_2_ activity. It is noteworthy that an increase of PLA2 activity by THC has been shown in homogenate of sea urchin sperm by Chang *et al.* [76]. The possible preferential localization of NAEs in specific domains, with different lipid composition, could give rise to microenvironments with different structural and physicochemical features [74,75,77] which could affect the activity of membrane enzymes, such as those involved in capacitation process [68] and calcium channels (which modulates HA) in men with low sperm kinematic parameters [67]. 

A PEA protective action against ROS cannot be excluded, in fact its antioxidative activities were shown in low-density lipoproteins [78] and in rat liver mitochondria [79]. Controlled generation of ROS has physiologic roles in important functions involved in fertilization, such as HA, capacitation and acrosome reactions [80]. However, increased levels of ROS were demonstrated in semen samples of infertile men [81] such as patients with idiopathic infertility [80] , suggesting that ROS-induced damages of spermatozoa, in particular, lipid peroxidation of sperm membrane, could be one of the key mechanisms involved in the pathophysiology of male infertility [80]. Because uncontrolled and excessive ROS concentration has a significant role as one of the major factors leading to the infertile status [80], it is reasonable to expect that it causes alteration of sperm physiologic functions, such as HA. In a previous work we have shown that addition of OEA (another NAE with antioxidant properties) to *in vitro* capacitated spermatozoa improves sperm kinematic parameters and HA, both in the presence and in the absence of oxidative stress, conferring, in some measure, protection against oxidative damage. We suggest that patients with idiopathic infertility, who suffer oxidative stress by increased levels of ROS [79] and need assisted reproduction procedures, could benefit from OEA *in vitro* supplementation during the preparation of their sperm cells for fertilization.

## 4. Conclusions

Many studies have been performed on the effects of endocannabinoids, such as AEA and 2-AG, on spermatozoa from different mammalian and non-mammalian species. Extensive and recent reviews have described [3,9,18,50,55,56] their effects on spermatozoa functions. All these papers indicate an inhibitory effect of these endocannabinoids on many fundamental sperm functions, although no precise target of these molecules was identified. This review presents some experimental data on PEA and OEA, endocannabinoid-like molecules, which appear to exert their action exclusively on sperm cells with altered features, such as membrane characteristics and kinematic parameters. Their beneficial effects on these cells could suggest a possible pharmacological use of PEA and OEA on patients affected by some forms of idiopathic infertility.
